# Effects of polymorphisms in *CAPN1* and *CAST* genes on meat tenderness of Chinese Simmental cattle

**DOI:** 10.5194/aab-61-433-2018

**Published:** 2018-11-02

**Authors:** Xiaomei Sun, Xiuxiang Wu, Yongliang Fan, Yongjiang Mao, Dejun Ji, Bizhi Huang, Zhangping Yang

**Affiliations:** 1 Key Lab of Animal Genetics, Breeding & Molecular Design of Jiangsu Province, Yangzhou University, Yangzhou 225009, China; 2 Joint International Research Laboratory of Agriculture and Agri-Product Safety of Ministry of Education of China, Yangzhou University, Yangzhou 225002, China; 3 Department of Neurobiology, Xuzhou Medical College, Xuzhou 221004, Jiangsu, China; 4 Academe of grassland and animal science, Kunming 650000, China

## Abstract

Considerable evidence has demonstrated that the μ-calpain (*CAPN1*) gene and its inhibitor calpastatin (*CAST*) gene are major factors affecting meat quality. Marker-assisted selection (MAS) has been widely used to improve beef quality traits. Therefore, the objective of the present study was to investigate the single nucleotide polymorphisms (SNPs) of bovine *CAPN1* and *CAST* genes using 367 animals representing the four main Chinese cattle breeds and to explore the effects of these SNPs on meat quality traits. Two SNPs within *CAPN1* and one SNP in *CAST* were successfully identified in cattle. Genetic diversity analyses suggested that most SNPs in the four breeds exhibited a moderate genetic diversity. Moreover, associations between individual markers and meat quality traits were analyzed in Chinese Simmental cattle. The *CAPN1* 4558 A > G locus was found to be significantly associated with shear force value (SFV) and marbling score (BMS), and *CAPN1* 4684 C > T exerted a significant effect on SFV, while the *CAST* genotype was not significantly associated with any of the measured traits. SFV, commonly used to measure meat tenderness, represents an important quality trait as it contributes to the flavor of cooked meat. This work confirms the effect of *CAPN1* on beef tenderness and lays an important foundation for future cattle breeding.

## 1 Introduction

Marbling and meat color are important meat quality traits that are usually used as an indicator of freshness and hygienic beef products (Castro et al., 2016). In addition, tenderness is considered as the most momentous parameter regarding eating quality (Moeller et al., 2010; Koohmaraie and Geesink, 2006; Kamruzzaman et al., 2013). Thus, color, marbling, tenderness, and flavor contribute importantly to various aspects of beef quality. Currently, consumer demand for high-quality meat is increasing, but the breeding and production of high-quality beef cattle cannot keep pace with it. Meat quality traits are normally influenced by the interaction of many factors, including the animal genetics. Single nucleotide polymorphism markers that can be measured in one or more populations play a central role in modern genetic breeding (Davey et al., 2011; Coll et al., 2014; Melo et al., 2017; Renaville et al., 2015) and are important tools for beef cattle breeding (Li et al., 2013a; Sun et al., 2013; Buzanskas et al., 2017). Characterization of the genes affecting important economical traits and the availability of the bovine genome sequence have allowed researchers to identify the polymorphisms associated with phenotype.

**Table 1:** The details of four Chinese cattle populations.

Cattle breed	Abbr.	Species	Sampling location	Number
ChineseSimmental	Sim	Cross-breed of *Bos taurus*	Inner Mongoliaprovince	Male 132
Leiqiong cattle	LQ	*Bos indicus*	Qiongzhong, Hainan province	Female 74
Yunnan highpump	YNH	*Bos indicus*	Puer, Yunnan province	Male 5,female 75
BMY cattle	BMY	Cross-breed of *Bos indicus*	Kunming, Yunnanprovince	Male 4, female 77

In cattle, genetic markers affecting meat color and beef marbling have rarely been studied, suggesting that the research is urgently needed. Several candidate genes have now been nominated with significant effects on meat tenderness. These include the protease μ-calpain (*CAPN1*) and its inhibitor calpastatin (*CAST*) (Barendse et al., 2007; Tait et al., 2014; Gandolfi et al., 2011; Schenkel et al., 2006). The *CAPN1* gene, encoding the larger subunit of the cysteine related to the postmortem tenderization process, was located on BTA29, which contains quantitative trait loci of meat quality. *CAST*, the inhibitor calpastatin of *CAPN1* (Koohmaraie, 1996), was also identified as a major factor affecting postmortem tenderization in meat (Enriquez-Valencia et al., 2017). Genetic marker tests for these proteins have been identified for meat tenderness and are now commercially available (Johnston and Graser, 2010). Some genetic markers with positive effects on certain traits are allowed to be incorporated into multi-trait breeding projects.

Discovery of the DNA markers associated with meat quality traits is becoming an interest in cattle breeding. Hence, in this study, *CAPN1* and *CAST* were selected as candidate genes to estimate the frequency of single nucleotide polymorphisms (SNPs) in four Chinese cattle breeds and to determine if individual SNPs were associated with meat quality traits in Chinese Simmental cattle. The results provide potential useful genetic information for beef quality trait improvements.

## 2 Materials and methods

### 2.1 Samples and meat quality traits

Genomic DNA samples (n=367) were collected from four Chinese cattle breeds: Chinese Simmental cattle (Sim, n=132), Leiqiong cattle (LQ, n=74), Yunnan high pump (YNH, n=80), and BMY cattle (1/2 Brahman, 1/4 Murray Grey, and 1/4 Yunnan Yellow cattle, n=81). All experiments performed in this study were approved by the Yangzhou University Animal Care and Use Committee (SYXK(SU)2017-0044). The care and use of experimental animals fully complied with local animal welfare laws, guidelines, and policies. Detailed information about the four populations is presented in Table 1. Four cattle breeds were used to investigate the genetic diversity of *CAPN1* and *CAST* genes. The Chinese Simmental cattle, a cross-bred beef cattle, was selected for evaluating the effect of *CAPN1* and *CAST* polymorphisms on meat quality traits and 132 muscle samples were collected from the Chinese Simmental cattle. Four meat quality traits, including shear force value (SFV), marbling score (BMS), meat color score (MCS), and fat color score (FCS), were recorded form 132 Chinese Simmental individuals.

Warner–Bratzler shear force values (SFVs) is one of the standard tools to quantify meat tenderness. SFV was measured on the interface of the longissimus muscle from the 12th and 13th ribs after 24–48 h of aging. The muscle samples were sliced into 2.5 cm thickness parallel to the longitudinal orientation of the muscle fibers and heated in a water bath until the center temperature reached 75 ${}^{\circ }$C. After chilling, six cores should be obtained from each sample. Round cores should be 1.27 cm in diameter and removed parallel to the longitudinal orientation of the muscle fibers so that the shearing action is perpendicular to the longitudinal orientation of the muscle fibers. Samples that are not uniform in diameter or have obvious connective tissue defects would not be representative of the sample and should be discarded. Muscle samples that meet the requirement were then measured for shear force values immediately using an XL1155 shear force detector (Xielikeji Co., Ltd, Harbin, China). Beef marbling score (BMS) was evaluated at the longissimus muscle of the 12th and 13th ribs using photographic standards.

### 2.2 SNP identification and genotyping

Genomic DNA was extracted from the blood samples following the standard procedures (Sambrook and Russell, 2001). The quantity and quality of DNA were measured by a spectrophotometer at 260/280 nm using an Eppendorf BioPhotometer. Each genome DNA sample was diluted to 50 ng µL${}^{-1}$. The PCR–single strand conformation polymorphism (PCR-SSCP) method was applied to detect the SNPs of two genes. Primers used for SNP identification and genotyping were synthesized by Shanghai Sangon (Table S1 in the Supplement). PCR amplification was performed using a GeneAmp PCR system 9700 thermal cycler (Applied Biosystems, Foster City, CA, USA), with the following protocol: initial denaturation at 94${}^{\circ }$ for 5 min, followed by 30 cycles at 94${}^{\circ }$ for 30 s, 65${}^{\circ }$ for 30 s, and 72${}^{\circ }$ for 40 s, with a final extension at 72${}^{\circ }$ for 7 min.

A total of 2.0 µL PCR product was mixed with 8 µL of the denaturation solution (50 mmol L${}^{-1}$ NaOH, 1 mmol L${}^{-1}$ EDTA) and 1 µL of the loading buffer containing 0.25 % bromophenol blue and 0.25 % xylene cyanol, denatured for 10 min at 98${}^{\circ }$, and rapidly chilled to -20${}^{\circ }$. The samples were then detected in 12 % sodium dodecyl sulfate-polyacrylamide gel electrophoresis (SDS-PAGE). A thermostatically controlled refrigerated circulator was used to maintain constant temperature (4${}^{\circ }$) of the gels. The gels were run in the following conditions: preelectrophoresis under 250 V and 40 mA for 10 min; and 150 V and 24 mA for 8 h. The gels were then stained by Silver Stain (Kucharczyk Techniki Elektroforetyczne). The patterns of DNA bands were observed and photographed with the GDS7500 system (UVP). Three samples of each genotype were selected for sequencing with the ABI-PRISM3730 analyzer.

### 2.3 Statistical analysis

Allele and genotype frequencies were calculated using PopGene (Ver. 3.2) and deviations from Hardy–Weinberg equilibrium (HWE) were checked by chi-square test. The population genetic indexes including gene heterozygosity (He), effective allele numbers (Ne), and polymorphism information content (PIC) were calculated by Nei's method (Nei and Roychoudhury, 1974).

The data were analyzed with the SPSS software with the following fixed-effects model. Multivariate analysis of variance was applied to study the effect of genotypes on meat quality traits within Sim cattle (n=132) using the general linear model (GLM), with genotype serving as fixed effects and BMS, SFV, MCS, and FCS as dependent variables:

1 $$Y=\mu +G_{j}+e,$$

where y represents the phenotype records of meat quality traits (e.g., the IMF, BMS, SFV, MCS, and FCS), μ is general mean, $G_{j}$ is the fixed effect of the genotype (*CAPN1* 4558, *CAPN1* 4684, and *CAST* 596), and e is the random error. The statistics were presented as probability values and least square means ± standard error, and P values < 0.05 were considered statistically significant.

## 3 Results

### 3.1 SNP identification and genotyping

**Figure 1:**
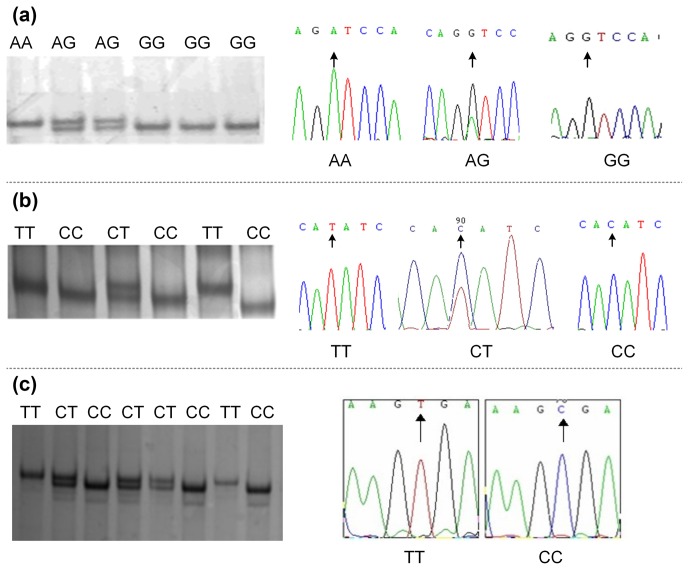
SNP identification and genotyping in bovine *CAPN1* and *CAST* genes by the PCR-SSCP method. **(a–c)** Electrophoresis patterns and sequencing results of *CAPN1* 4558 A > G, *CAPN1* 4684 C > T, and *CAST* 596 T > C, respectively.

Polymorphisms were detected by DNA pool sequencing. Investigation of exon 1 to exon 6 of the bovine *CAPN1* gene and exon 9 of the *CAST* gene identified three SNPs, and they were located in exon 2 and exon 4 of *CAPN1* and exon 9 of *CAST*, respectively. These SNPs, including *CAPN1* 4558 A > G, *CAPN1* 4684 C > T, and *CAST* 596 T > C, were successfully genotyped by PCR-SSCP (Fig. 1a, b, and c). All SNPs contained two alleles and three genotypes in each population.

### 3.2 Genetic diversity analyses among four Chinese cattle populations

Genetic diversity analyses are given in Table 2. Allele frequencies, genotype frequencies, and PIC at all SNPs were similar in LQ and YNP, two *Bos indicus* cattle breeds.

At the *CAPN1* 4558 A > G locus, the A allele was predominant in all populations, with frequencies ranging from 0.664 to 0.889 within the four beef breeds (Sim, LQ, YNP, and BMY). At the *CAPN1* 4684 C > T locus, the C allele was predominant in Sim, LQ, and YNP with frequencies varying from 0.516 to 0.818, while the frequency of the C allele in BMY was 0.470. At the *CAST* 596 T > C locus, the T allele was predominant in all populations, with frequencies ranging from 0.529 to 0.626 within the four beef breeds.

PIC analysis suggested that at the *CAPN1* 4684 C > T and *CAST* 596 T > C loci, all four breeds had a moderate genetic diversity (0.250 < PIC < 0.500). At *CAPN1* 4558 A > G loci, the Sim breed had a moderate genetic diversity, while LQ, YNP, and BMY had a low genetic diversity (PIC < 0.250). Moreover, the χ2 test showed that the genotype distributions of all SNP loci within Chinese Simmental cattle breed and most SNP loci in the other three breeds were in HWE (P>0.05, Table 2). This indicated that the four Chinese cattle populations, especially the Chinese Simmental cattle breed, have experienced low selection pressure and therefore may have great genetic potential in cattle breeding.

**Table 2:** Genotype, allele frequencies, and genetic diversity parameters of bovine *CAPN1* and *CAST* in four Chinese cattle populations.

Loci	Breeds	Genotypic frequencies			Allelic frequencies		$\chi^{2}$ (HWE)${}^{\text{a}}$	Diversity parameter${}^{\text{b}}$		
		GG	GA	AA	G	A		He	Ne	PIC
*CAPN1* A4558G	Sim(132)	0.117	0.438	0.445	0.336	0.664	0.400	0.446	1.806	0.347
	LQ(74)	0.014	0.274	0.712	0.151	0.849	19.450**	0.256	1.345	0.224
	YNP(80)	0.050	0.262	0.688	0.184	0.816	0.661	0.230	1.429	0.255
	BMY(81)	0.074	0.074	0.852	0.111	0.889	0.294	0.197	1.246	0.178
		CC	CT	TT	C	T				
*CAPN1* C4684T	Sim(132)	0.252	0.528	0.220	0.516	0.484	0.048	0.499	1.998	0.375
	LQ(74)	0.479	0.233	0.288	0.600	0.400	0.366	0.482	1.930	0.366
	YNP(80)	0.667	0.282	0.051	0.808	0.192	1.010	0.310	1.450	0.262
	BMY(81)	0.240	0.460	0.300	0.470	0.530	31.640**	0.498	1.993	0.374
		TT	TC	CC	T	C				
*CAST T596C*	Sim(132)	0.112	0.523	0.365	0.374	0.626	1.486	0.464	1.881	0.359
	LQ(74)	0.324	0.284	0.392	0.466	0.534	12.741**	0.498	1.994	0.374
	YNP(80)	0.212	0.450	0.338	0.437	0.563	0.481	0.493	1.972	0.371
	BMY(81)	0.203	0.348	0.449	0.377	0.623	6.020*	0.470	1.886	0.359

Note: $\chi_{0.05}^{2}=5.001$, $\chi_{0.01}^{2}=9.21$; * means P<0.05, ** P<0.01. ${}^{\text{a}}$ $\chi^{2}$ (HWE), Hardy–Weinberg equilibrium $\chi^{2}$ value. ${}^{\text{b}}$ He, gene heterozygosity; Ne, effective allele numbers; PIC, polymorphism information content.

**Table 3:** Association analysis between SNP effects and meat quality traits in Chinese Simmental cattle.

Loci	Genotypes	SFV (kg)	BMS	MCS	FCS
		(mean±SE)	(mean±SE)	(mean±SE)	(mean±SE)
*CAPN1* A4558G	GG (32)	3.89±1.21${}^{\text{a}}$	5.57±0.65${}^{\text{a}}$	4.86±0.66	3.79±0.58
	AG (67)	4.71±1.70${}^{\text{b}}$	5.89±0.32${}^{\text{b}}$	4.98±0.87	3.47±0.63
	AA (28)	4.51±1.49${}^{\text{b}}$	5.95±0.23${}^{\text{b}}$	4.89±0.81	3.55±0.63
*CAPN1* C4684T	CC(15)	4.51±1.37${}^{\text{ab}}$	5.90±0.31	4.93±0.74	3.43±0.68
	CT(56)	4.81±1.63${}^{\text{a}}$	5.86±0.39	4.98±0.87	3.58±0.61
	TT(57)	4.09±1.54${}^{\text{b}}$	5.89±0.32	4.89±0.89	3.48±0.64
*CAST* T596C	TT(12)	4.57±1.92	5.82±0.41	4.64±0.81	3.73±0.47
	CT(74)	4.56±1.52	5.88±0.37	4.97±0.89	3.50±0.63
	CC(39)	4.76±1.63	5.89±0.31	5.00±0.81	3.53±0.65

Note: ${}^{\text{a-b}}$ Different letters indicate significant difference between genotypes, p<0.05. BMS, marbling score; SFV, shear force value; MCS, meat color score; FCS, fat color score.

### 3.3 Association of genotype with meat quality traits in Chinese Simmental cattle

The relationships between the *CAPN1* and *CAST* genotypes and meat quality traits were analyzed in Chinese Simmental cattle (n=132). Traits evaluated were shear force value (SFV), marbling score (BMS), meat color score (MCS), and fat color score (FCS). The results are exhibited in Table 3. At the *CAPN1* 4558 A > G locus, cattle with the GG genotype displayed lower shear force value than individuals with AG and AA genotypes (P<0.05). At the *CAPN1* 4684 C > T loci, animals with the TT genotype had significantly lower shear force value than those with the CC and CT genotype (P<0.05). In addition, the *CAPN1* 4684 C > T locus was found to be significantly association with BMS (P<0.05), while the *CAST* 596 T > C loci had no significant association with any of the measured traits (P>0.05).

## 4 Discussion

Consumers are increasingly aware of dietary nutrition, and beef is considered to be a highly nutritious food that contains biological value protein and micronutrients (Scollan et al., 2014). Thus, these trends are also occurring in the fresh beef market as reflected by the demand for higher-quality meat. Meat quality is difficult to define because it is a complex concept affected by various intrinsic qualities (for example, color, tenderness, juiciness, flavor, chemical composition) and extrinsic qualities (brand, quality mark, production environment) (Joo et al., 2013). Knowledge of the above factors has augmented consumer interest in food quality. Beef tenderness is a critical component of palatability, but the difficulty in obtaining phenotypic data until slaughter has made it hard to select for this trait. Therefore, marker-assisted selection (MAS) for genetic improvement could bypass this obstacle if appropriate markers could be found. Thus, increasing attention has been given to improving beef quality traits through MAS.

Considerable evidence has demonstrated that allelic variation in the *CAPN1* gene is important in the successful application of MAS in beef cattle populations (Schenkel et al., 2006). It plays an important role in meat tenderness and indirectly acts on other meat quality traits. In the same process, *CAST* appears to be an inhibiting enzyme of *CAPN1*. In beef cattle breeding, genetic variation of meat tenderness, structure, and color plays important roles in the MAS (Lee et al., 2014). Therefore, acquiring a highly efficient system for identifying the molecular markers of these genes would constitute an important support in the selection of cattle to produce better beef.

In the present study, SNPs in *CAPN1* and *CAST* were identified and analyzed for associations with meat quality traits in Chinese Simmental cattle. Briefly, the presence of *CAPN1* markers had significant effects on beef shear force but no detectable effects were found for the *CAST* SNP, which agrees with a previous study on beef cattle (Corva et al., 2007). Tenderness, measured by shear force, represents a vital beef quality trait as it contributes to the flavor of cooked meat (Kamruzzaman et al., 2013). Several research studies have been applied to the effects of genetic markers in *CAPN1* on beef tenderness (Allais et al., 2011; Curi et al., 2009). In addition, previous studies showed that SNPs in the *CAPN1* gene were associated with the level of beef marbling (Li et al., 2013b). Our data illustrated that *CAPN1* 4558 A > G was found to be significantly associated with BMS and SFV, and *CAPN1* 4684 C > T was found to exert a significant effect on SFV in Chinese Simmental cattle (* p<0.05). Hence, these genetic markers may be useful in selecting populations for beef breeding.

As a representative of the calpain system, *CAST* was recognized as a candidate marker in beef breeding with MAS. Herein, no significant association between identified SNPs of the *CAST* gene and meat quality traits was found in the Chinese Simmental cattle population. This result was consistent with previous research performed on beef cattle from Argentina and on sheep (Corva et al., 2007; Zhou et al., 2008), but disagreed with some research showing that the *CAST* genotype was associated with meat tenderness in other cattle populations (Casas et al., 2006; Allais et al., 2011; Calvo et al., 2014; Enriquez-Valencia et al., 2017), indicating that this SNP may not be a powerful marker for meat quality traits in Chinese Simmental cattle.

## 5 Conclusions

In conclusion, this study supports previous work that *CAPN1* genetic markers have a positive effect on meat tenderness and BMS traits, the most important meat quality traits for consumers. The associations between the genotyped SNPs in this research and meat quality traits are of potential interest to the beef industry and could lay an important foundation for the expanding panel of functional variation relevant to meat quality.

## Supplement

10.5194/aab-61-433-2018-supplement The supplement related to this article is available online at: https://doi.org/10.5194/aab-61-433-2018-supplement.

## Data Availability

Data are available upon reasonable request from the corresponding author.
