# Supplementary material for: Effects of polymorphisms in CAPN1 and CAST genes on meat tenderness of Chinese Simmental cattle
Source: Arch Anim Breed. 2018 Nov 2;61(4):433–9. doi: 10.5194/aab-61-433-2018 (PMC7065412; doi:10.5194/aab-61-433-2018)
Supplement: The supplement related to this article is available online at: https://doi.org/10.5194/aab-61-433-2018-supplement. [file aab-61-433-supplement.pdf]

Supplement of Arch. Anim. Breed., 61, 433–439, 2018
https://doi.org/10.5194/aab-61-433-2018-supplement
© Author(s) 2018. This work is distributed under
the Creative Commons Attribution 4.0 License.

Open Access
Archives Animal Breeding

Supplement of

Effects of polymorphisms in *CAPN1* and *CAST* genes on meat tenderness of Chinese Simmental cattle

Xiaomei Sun et al.

Correspondence to: Zhangping Yang (yzp@yzu.edu.cn)

The copyright of individual parts of the supplement might differ from the CC BY 4.0 License.

Table S1. The primers used for PCR–SSCP analysis of bovine *CAPN1* and *CAST* genes

Primer List	Gene	Position	Primer sequence (5'-3')	Accession Number	Size (bp)	T_m (° C)
P1	CAPN1	Exon 1	F:GGCCGAGGAGATACCGTGAA<br>R:TTACCTGGGGGAGGAGTGTTG	AF248054	222	61.2
P2	CAPN1	Exon2	F:CCTTCCTCGTTCCTTGTGCTA<br>R:CCCAAGCCTGTGGTTTCC	AF248054	246	58.6
P3	CAPN1	Exon3	F:TGACTTTGTGCTGCGTTTCT<br>R:AGACCAAGACACAGGACACCC	AF248054	247	58.6
P4	CAPN1	Exon4	F:CAATCTCCCCGACGAGGT<br>R:CCGGGTGATCCAGGTAAACAG	AF248054	244	58.6
P5	CAPN1	Exon 5	F:ATAGAGGCTGGGCAGGTCAGT<br>R:AACCAGGATGCCCAGTCG	AF248054	238	55
P6	CAPN1	Exon 6	F:GGATCTCTGGTTTCTGAGGGT<br>R:AGGGCATAGAGAGCAGTC	AF248054	229	53.2
P7	CAST	Exon9	F：TGGGGTGTATGCGTGTTT<br>R：TCCAATCCTGCACTCCTG	AF158246	257	54.2

Note: Tm, annealing temperature
